# Ocular Inflammation Post-Vaccination

**DOI:** 10.3390/vaccines11101626

**Published:** 2023-10-23

**Authors:** Yaru Zou, Koju Kamoi, Yuan Zong, Jing Zhang, Mingming Yang, Kyoko Ohno-Matsui

**Affiliations:** Department of Ophthalmology & Visual Science, Graduate School of Medical and Dental Sciences, Tokyo Medical and Dental University, Tokyo 113-8510, Japan; alicezouyaru519@gmail.com (Y.Z.); zongyuan666.oph@tmd.ac.jp (Y.Z.); zhangjing.oph@tmd.ac.jp (J.Z.); yangmm12.oph@tmd.ac.jp (M.Y.); k.ohno.oph@tmd.ac.jp (K.O.-M.)

**Keywords:** vaccine, post-vaccination, immunization, ocular complications, ocular side effects, uveitis

## Abstract

The association between vaccines and ocular disorders has attracted significant attention in scientific research. Numerous mainstream vaccines are associated with a range of uveitis types, including anterior, intermediate, and posterior uveitis. Additionally, they are associated with distinct ocular diseases such as multifocal choroiditis, Vogt–Koyanagi–Harada (VKH) disease, acute posterior multifocal placoid pigment epitheliopathy (APMPPE), and multiple evanescent white dot syndrome (MEWDS). These ocular conditions are often transient, with a vast majority of patients experiencing improvement after steroid intervention. To date, numerous cases of vaccine-induced uveitis have been reported. This study analyzed the correlation between antiviral vaccines, including the hepatitis B virus (HBV), human papillomavirus (HPV), measles–mumps–rubella (MMR), varicella zoster virus (VZV), and influenza vaccines, and different manifestations of uveitis. This is the first comprehensive study to offer a detailed analysis of uveitis types induced by antiviral vaccines. Through an extensive database search, we found a particularly strong link between influenza vaccines, followed by VZV and HPV vaccines. While anterior uveitis is common, conditions such as APMPPE, MEWDS, and VKH are particularly notable and merit careful consideration in clinical practice. Corticosteroid treatment was effective; however, half of the observed patients did not achieve full recovery, indicating potentially prolonged effects of the vaccine.

## 1. Introduction

Vaccines play a pivotal role in the prevention and control of infectious diseases and significantly affect global health [1]. By preventing the spread of infectious diseases and reducing the burden on health-care systems, vaccines can save countless lives and improve community well-being. According to World Health Organization (WHO) data, immunization currently prevents 3.5–5 million fatalities annually by targeting diseases such as pertussis, influenza, measles, diphtheria, and tetanus [2]. As biological agents, vaccines contain attenuated or inactivated pathogens or their derivatives, prompting the immune system to produce antibodies, memory cells, and other immune responses that recognize and target specific pathogens [3]. Vaccines can be classified into two categories based on the type of pathogen: (a) viral (b) bacterial. A more detailed classification includes live attenuated vaccines, such as the measles–mumps–rubella (MMR) vaccine [4]; inactivated vaccines, such as influenza and hepatitis A vaccines; subunit vaccines, such as recombinant hepatitis B virus surface antigen vaccines and adjuvants [5,6]; and mRNA vaccines, such as those developed for COVID-19 [7].

Vaccines offer significant benefits, but also have potential side effects. Uveitis and ocular inflammation are relatively prevalent among these complications [8], with annual post-vaccine uveitis incidence ranging from 8 to 13 per 100,000 cases/year [9]. Uveitis involves inflammation of the uvea and the middle layer of the eye, including the iris, ciliary body, and choroid. Based on its anatomical location, it can be classified as anterior, intermediate, posterior, or pan-uveitis [10]. Uveitis often has a chronic relapsing course, leading to temporary or permanent vision impairment, making it one of the leading global causes of blindness [11]. According to the etiology, uveitis can be divided into two categories: infectious and noninfectious. Infectious uveitis includes bacterial, fungal, viral, spirochete, and parasitic diseases; infectious uveitis includes idiopathic, traumatic, autoimmune, and rheumatic immune diseases [12]. Viruses are a major cause of infectious uveitis. Half of the human herpes viruses, including herpes simplex virus (HSV) [12,13], varicella zoster virus (VZV) [14], and cytomegalovirus (CMV) [15], account for 5–10% of all uveitis cases [16,17,18]. These include rubella virus [19] and human T-lymphotropic virus 1 (HTLV-1) [20]. However, there is no clear evidence of a causal link between antiviral vaccination and uveitis. This study aimed to review the literature on the possible relationship between uveitis and antiviral vaccines.

## 2. Methods of Literature Search

The Medline [PubMed], Embase, and Cochrane Library databases (from 1983 to June 2023) were used to search the relevant publications that included case reports and series. All the sourced articles had their full text reviewed to ensure that the contents were relevant to the study.

## 3. Results

### 3.1. General Analysis Regarding Cases of Uveitis Post-Vaccination

After conducting a comprehensive review, a total of 51 reported cases (involving 61 patients), spanning from 1978 to 2023, have been associated with uveitis following various vaccinations [21,22,23,24,25,26,27,28,29,30,31,32,33,34,35,36,37,38,39,40,41,42,43,44,45,46,47,48,49,50,51,52,53,54,55,56,57,58,59,60,61,62,63,64,65,66,67,68,69,70,71] (Table 1), including 4 case reports (5 patients) related to hepatitis B virus (HBV) vaccination [28,29,30,31]; 7 case reports (8 patients) related to human papillomavirus (HPV) vaccination [21,22,23,32,33,34,35]; 16 cases (18 patients) regarding uveitis after influenza vaccination [36,37,38,39,40,41,42,43,44,45,46,47,48,49,50,51]; 3 cases (4 patients) related to measles–mumps–rubella (MMR) vaccination [52,53,54]; 12 cases (16 patients) related to varicella zoster virus (VZV) vaccination [24,25,26,27,55,56,57,58,59,60,61,62]; 4 cases (5 patients) for yellow fever [63,64,65,66]; one each for hepatitis A virus (HAV) [67] and rabies virus vaccination [68]; and 3 case reports involving mixed vaccine administrations [69,70,71].

The average age of the affected individuals was 38.5 years (range, 1–86 years), with a female predominance (female:male = 35:26). Based on the site of infection, this study included 11 cases of anterior uveitis [27,35,37,45,52,54,56,58,63], 1 case of intermediate uveitis [63], 8 cases of posterior uveitis [21,22,27,28,32,42], 3 cases of uveitis involving both anterior and intermediate segments [27,55,64], 1 case of anterior and posterior uveitis [38], and 7 cases of pan-uveitis [23,34,40,43,48,53]. Distinct types of uveitis, such as acute posterior multifocal placoid pigment epitheliopathy (APMPPE) [36,41,46,47,57], Vogt–Koyanagi–Harada syndrome (VKH) [31,39,49,51,65,66], multiple evanescent white dot syndrome (MEWDS) [30,33,50,67,68,69,70], acute retinal necrosis (ARN) [24,25,26,59,61,62], and uveitis sarcoidosis [60] were also included. Most patients presented with bilateral involvement (bilateral:unilateral = 36:25), and left eye involvement was more common among patients with unilateral involvement (left:right = 16:9). In addition to cases lacking specific timeframes, uveitis typically occurs within days to years after vaccination (range, 24 h to 3 years), with some cases showing symptoms within 24–48 h, suggesting a potential association between vaccines and uveitis.

Of the reported cases, 32 patients presented systemic symptoms [24,27,28,31,33,34,35,36,37,38,39,41,42,45,46,47,48,49,50,51,53,55,56,57,60,63,64,65,66,71]. Most patients achieved full recovery following anti-inflammatory medications, intravenous and/or oral corticosteroids, intraocular injections, and periocular administration. However, it is noteworthy that a subset of patients exhibited varying degrees of residual symptoms even after systematic and targeted treatment during the follow-up period (range, 1 month to 5 years).

### 3.2. Detailed Analysis by Vaccine

#### 3.2.1. Human Papillomavirus (HPV) Vaccine

HPV is a sexually transmitted virus that, if persistently infected with high-risk genotypes (such as HPV 16 and 18), is strongly associated with cervical cancer [72,73]. Currently, there is no virus-specific treatment for HPV infections; however, preventive measures are available. Six prophylactic HPV vaccines have been licensed using recombinant DNA and cell culture techniques to produce purified L1 structural proteins that self-assemble into virus-like particles (VLPs) [74]. As of the end of 2022, the estimated global coverage rate for the first dose of HPV vaccination among girls was 21% [75]. It is noted that all types of HPV vaccines should be used in females aged 9 and above. They are licensed for use by people up to the age of 26 or 45 [76]. As awareness of HPV vaccines grows among women, monitoring of adverse reactions should also increase.

**Figure 1:**
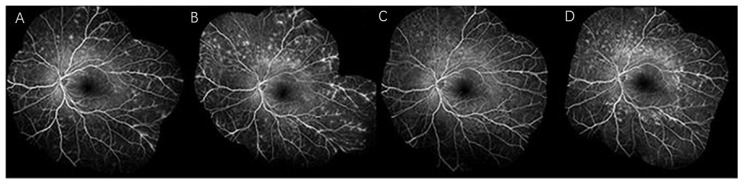
Fundus images of a patient with multiple evanescent white dot syndrome after human papillomavirus vaccination. FA revealed leakage from the mid-peripheral vasculature at 7 months (**A**), FA was performed again and revealed a marked increase in vascular leakage at 1.5 years (**B**), 1 week after steroid pulse therapy, FA showed a drastic reduction in leakage from the vasculature (**C**), FA revealed recurrent leakage after 2 years (**D**) (adapted with permission from [33] 2014, Ogino et al.).

By 2023, numerous adverse events related to HPV have been reported, including autoimmune diseases such as scleroderma and rheumatoid arthritis [77]. However, ocular side effects are limited. Our study specifically focused on uveitis and compiled the reported cases of HPV-related uveitis to date (Table 1). Seven cases involving eight female patients (average age = 21.75 years) [21,22,23,32,33,34,35] developed uveitis at various locations following HPV vaccination (two anterior [35], one posterior [32], two pan-uveitis [23,34], one MEWDS [33], and two Harada-like uveitis [21,22]). Symptoms manifested within weeks, ranging from 4 days to 10 weeks after vaccination, with the majority (four out of eight) occurring after the third vaccine dose [21,22,34,35]. Except for one unreported case [35], approximately 42.9% (three out of seven) of patients exhibited systemic symptoms such as fever, hearing loss, sore throat, headache, and joint pain [33,34,35]. With an average follow-up period of 1.2 years, vision completely recovered in five patients following corticosteroid treatment [21,22,23,34,35], including one patient who recovered with eyedrops alone [21]. However, some patients experience prolonged effects, such as recurrent leakage [33] (Figure 1) and extensive macular scarring [32]. Furthermore, one patient required long-term medication to manage uveitis [35]. These enduring side effects are likely to persist throughout a patient’s lifetime and should therefore be treated with the utmost seriousness.

Three patients developed Harada-like uveitis [21,22,23], two of whom tested positive for HLA-DRB1*0405 [23] and DR04 and 07 suballeles [22]. In a recent meta-analysis, the odds ratios (OR) of developing VKH was 10.3 for HLA-DRB1*0405 [78]. However, rapid and significant recovery after a relatively short course of corticosteroid treatment is an atypical finding in Harada’s disease, which typically requires corticosteroid immunosuppressive therapy for at least 3–6 months [79]. Their clinical course more closely resembles that of vaccine-induced uveitis than that of coincidental autoimmune diseases. Tubulointerstitial nephritis and uveitis (TINU) syndrome is an autoimmune disorder widely suspected to be triggered by infections or drug hypersensitivity reactions [80]. Two patients with TINU syndrome following HPV vaccination have been reported [35], who exhibited clinical manifestations of interstitial nephritis, including glucosuria, proteinuria with low molecular weight, and elevated serum creatinine levels, and subsequently developed eye-related symptoms, such as redness and photophobia. Given the similarities between these two cases and their HPV vaccination histories, it is speculated that this could be related to HPV vaccine administration.

The WHO guidelines have acknowledged a “possible” association between HPV vaccination and uveitis [81]. Potential mechanisms include broad nonspecific immune responses or molecular mimicry. Comparative computer analysis of HPV 16 oncogenic proteins and human self-proteins has revealed molecular mimicry and demonstrated high and widespread similarity in numerous critical regulatory processes. Causality assessment is typically based on data regarding the administration, development of uveitis, and timing of rechallenge. However, existing case reports do not definitively establish a causal relationship and necessitate further investigation of the underlying mechanisms [82].

Considering these findings, health-care practitioners should be vigilant about the potential association between HPV vaccination and uveitis. When obtaining medical histories from patients with suspected uveitis, detailed inquiries about vaccination history and extraneous ocular manifestations should be made. Intravitreal injections may serve as a potential therapeutic option, especially for patients with contraindications to systemic corticosteroids. Extended follow-ups and proactive interventions are recommended for patients at risk of long-term adverse reactions.

#### 3.2.2. Hepatitis B Virus (HBV) Vaccine

As a DNA virus [83], HBV is the most common cause of liver cancer worldwide [84]. The widespread adoption of the hepatitis B vaccine has significantly reduced the global prevalence of the virus [85]. By the end of 2022, hepatitis B vaccination for infants had been implemented nationwide in 190 member countries. The global coverage for the three-dose hepatitis B vaccine was estimated to reach 84% [75]. This vaccine is produced through recombinant DNA technology, utilizing purified HBsAg antigen, and is typically administered in three doses: within 24 h of birth, at 1 month, and at 6 months [86].

Research indicates that uveitis is the most frequent ocular complication following HBV vaccine administration [87]. Our study encompassed four reports (involving five patients) of HBV-related uveitis [28,29,30,31], ranging in age from 20 to 43 years, with a male-to-female ratio of 2:3. The ocular lesions were bilateral and included two patients diagnosed with APMPPE [29], one with MEWDS [30], one with VKH syndrome [31], and one with bilateral posterior uveitis [28]. Therefore, HBV vaccine-induced uveitis presents with diverse manifestations, primarily bilateral and posterior uveitis. Some patients report systemic symptoms such as headaches, hearing loss, and skin changes [28,31]. The average duration was 4.8 days (range, 1–14 days). During the follow-up period ranging from 3 to 9 months, two patients still exhibited residual paracentral scotomas and potential persistence [29], while one patient responded to oral and topical steroids, but required prolonged treatment [31].

A potential mechanism underlying the development of uveitis may involve a hypersensitivity reaction. Fried et al. speculated that the vaccine surface forms immune complexes with hepatitis B antibodies, leading to uveitis [28]. While there is an ongoing debate about the role of this mechanism [88,89], evidence suggests immune complex deposition diseases, such as cryoglobulinemia and glomerulonephritis, during chronic hepatitis B infection [90,91,92]. Aguirre et al. conducted a study reinforced this perspective by discovering type III hypersensitive antigen-antibody complexes in the aqueous humor of dogs after adenovirus vaccination [90]. Another potential mechanism may involve the adjuvant effects of the vaccine vectors. Adjuvants, which are often combined with vaccines to enhance immunogenic activity, have been implicated in the development of uveitis in animal models [93]. Other autoimmune diseases, including multiple sclerosis and dermatomyositis, have been reported after hepatitis B vaccination, with a potential role in the molecular mimicry between retinal pigment epithelial protein and hepatitis B surface antigen [94,95,96,97,98]. A case report concerning the development of APMPPE after immunization with a recombinant hepatitis B virus vaccine suggested that the hepatitis B surface antigen may trigger immune-mediated retinal pigment epithelium disruption or choroidal vascular occlusion [29]. To some extent, these mechanisms contribute to establishing a causal link between hepatitis B vaccination and uveitis.

In summary, patients typically develop uveitis-related ocular symptoms shortly after vaccine administration. Clinicians should be attentive to such cases and consider uveitis-related examinations and targeted treatments for affected individuals.

#### 3.2.3. Influenza Virus Vaccine

Influenza is a widespread febrile illness that causes millions of severe cases, resulting in 290,000 to 650,000 deaths annually [99]. Vaccination is a pivotal strategy for preventing and controlling the spread of influenza A and B viruses, which are the primary causes of human seasonal influenza [100]. According to statistics, vaccination coverage for seasonal influenza among high-risk groups varies significantly across countries. The vaccination rates for chronic disease patients and health-care workers in most countries fall below 40% [101]. Various types of influenza vaccines, including live attenuated influenza vaccines (LAIV4), inactivated influenza vaccines (IIV4s), and recombinant influenza vaccines (RIV4) are currently available [102]. Owing to continuous antigenic changes in influenza viruses, health authorities engage in annual surveillance to identify the strains most likely to cause illness in the upcoming flu season [103]. In general, influenza vaccines are safe and effective, with minor side effects, such as local swelling, joint pain, and low-grade fever. Severe adverse events are extremely rare. However, in recent years, ocular reactions attributed to influenza vaccination have been reported, including, reactivation of herpes simplex keratitis, corneal transplant rejection [104], “ocular respiratory syndrome” (conjunctivitis and respiratory symptoms) [105], and bilateral optic neuropathy [106]. Uveitis is a relatively common ocular manifestation of influenza vaccination.

**Figure 2:**
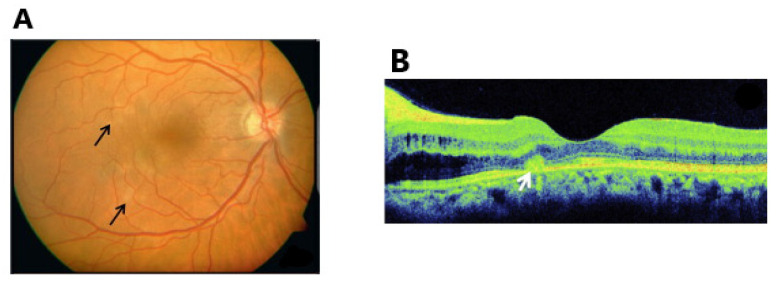
Fundus images of a patient with uveitis following influenza vaccination. Multiple white dots (black arrow) at the level of outer retina are observed in patient with MEWDS (**A**). A whitish spot (white arrow) found in optical coherence tomography in patient with APMPPE (**B**). ((**A**) Adapted with permission from [44] 2013, Goyal et al.; (**B**) Adapted with permission from [46] 2016, Gonome et al.).

Sixteen cases (involving eighteen patients) following influenza vaccination have been reported [36,37,38,39,40,41,42,43,44,45,46,47,48,49,50,51]. Most cases have been associated with live attenuated vaccines [36,42,43,45,48,49]. The final diagnoses varied and included conditions such as APMPPE [36,41,47,50], MEWDS [44,50], VKH disease [39,49,51], uveitis involving both anterior and posterior segments [38], anterior uveitis [37,45] and posterior uveitis [42] to pan-uveitis [39,40,43,48]. The sex distribution among the patients was balanced, with an average age of 42.7 years (range, 10–78 years), indicating that influenza vaccines may affect individuals of all ages without sex disparity. Nearly all the patients exhibited flu-like systemic symptoms. On average, ocular symptoms started 12.7 days after vaccination, with some cases manifesting within 24 h after the first dose [40]. When the initial flu-like symptoms fail to resolve, most patients are treated with topical or systemic corticosteroids and show significant improvement, suggesting that this is an autoimmune response rather than an infection. The follow-up period ranged from 1 month to 5 years, and 8 patients experienced significant symptom alleviation [38,39,40,42,44,45,46]. Patients with APMPPE and MEWDS often exhibit extensive white lesions on fundus examination and OCT, with residual retinal pigment epithelium (RPE) changes observed during long-term follow-up (Figure 2). Severe vision loss occurred in three patients, with one patient’s left eye remaining devoid of light perception even after one year of follow-up following systemic corticosteroid therapy [48]. Tao et al. reported the case of a 47-year-old female developed systemic fever, headache, flu-like symptoms, and ocular manifestations two days after vaccination [42]. The diagnoses included bilateral posterior uveitis and exudative retinal detachment (ERD). Although the exudative retinal detachment recovered after steroid therapy, visual acuity remained poor (0.01), possibly due to a prolonged disease course. Therefore, if not managed promptly and effectively, patients with suspected vaccine-induced uveitis may be at risk of blindness.

Uveitis can result from immune factors triggered by molecular mimicry between vaccine peptide fragments and retinal self-antigens, delayed hypersensitivity reactions [4,107,108]. It has been proposed that in live attenuated vaccines, inflammation might arise from viral peptide-induced reactions [4], whereas inactivated or subunit/conjugate vaccines can trigger inflammation from adjuvants, such as aluminum salts [107].

In summary, clinicians should monitor patients with a history of influenza vaccination who present with flu-like symptoms shortly after vaccination, and conduct comprehensive ocular examinations to assess the risk of uveitis. Absence of response to antibiotic treatment and negative test results. The absence of response to antibiotic treatment and negative test results can provide vital cues. Moreover, prompt initiation of judicious corticosteroid therapy upon diagnosis is imperative because delayed treatment may lead to adverse outcomes, including blindness. Considering the evolving composition of influenza vaccines each year to combat rapidly mutating viruses, we recommend annual influenza vaccination.

#### 3.2.4. Measles–Mumps–Rubella (MMR) Vaccine

The MMR vaccine, a combination of three live attenuated viruses, was licensed in 1971 [54]. Currently, 83% of children receive the first dose of measles vaccine before turning two, and 74% receive the second dose. The global coverage for the rubella vaccine is estimated at 68%, and the mumps vaccine has been implemented in 123 member countries worldwide [75]. The Advisory Committee on Immunization Practices recommends administration of the first dose at the age of 12–15 months and the second dose at 4–6 years. Rubella vaccine can be administered alone or in combination with measles and mumps vaccines [109], reducing the incidence of severe congenital rubella syndrome, which is characterized by deafness, ocular abnormalities, and congenital heart defects [110].

Our study included four cases of MMR vaccine-associated uveitis, with an average age of 11 years old and a male-to-female ratio of 3:1. Almost all the patients achieved complete recovery during the follow-up period after local or systemic steroid treatment. Surprisingly, case reports since 2004 have used polymerase chain reaction (PCR) and Goldmann–Witmer coefficient (GWC) analysis of anterior chamber fluid to identify a correlation between rubella virus (RV) and Fuchs’s heterochromic iridocyclitis syndrome (FHI) [16,111,112,113]. In a retrospective study involving 127 patients, all Fuchs’s heterochromic iridocyclitis cases were RV-positive except for two (5%) [113]. This aligns with a case report we collected [54], detailing a 12-month-old Caucasian girl who developed conjunctival redness, photophobia, heterochromia, and redness of the iris three months after receiving the MMR-attenuated live vaccine. Serological analysis revealed RV immunoglobulin G (IgG) positivity, underscoring the potential pathogenic role of the MMR vaccine.

Multiple factors may contribute to the development of severe uveitis following MMR vaccine administration: (a). Antigenic mimicry: evidence suggests that immune activation pathways induced by antigenic mimicry may lead to uveitis following administration of attenuated live MMR vaccine [52]. (b). Vaccine contamination: contamination of the vaccine with toxins, antigens, or vaccine modifications due to improper storage may play a role in uveitis induction. (c). Nature of attenuated live vaccines: the survival of viruses within attenuated live vaccines may contribute to the occurrence of uveitis. (d). Coincidence: although rare, the possibility of pure coincidence cannot be ruled out.

In conclusion, most patients achieved a complete recovery shortly after steroid treatment. However, refractory uveitis and other severe consequences may occur in specific instances. Our findings underscore the importance of increased ocular awareness following the MMR vaccination. Close monitoring, particularly among adolescents, is recommended for all recipients. Furthermore, for young patients exhibiting signs of Fuchs’s syndrome, such as heterochromia and posterior synechiae, aqueous humor examination is recommended to assess the risk of RV infection.

#### 3.2.5. Varicella Zoster Virus (VZV) Vaccine

VZV can cause herpes zoster following primary infection [114], primarily affecting individuals aged 70 years and older, potentially leading to severe complications such as postherpetic neuralgia [115]. These challenges underscore the compelling rationale for developing effective immunization strategies against VZV in the elderly population [116]. Subcutaneously administered VZV vaccines include Varivax (Merck, Whitehouse Station, NJ, USA), a live attenuated varicella vaccine for infants; Zostavax (Merck), approved for the secondary prevention of herpes zoster reactivation in individuals 75 years and older; and the recombinant subunit vaccine (RZV, Shingrix), introduced in 2017 [117].

Our study consists of 12 cases comprising 16 patients with VZV-associated uveitis [24,25,26,27,55,56,57,58,59,60,61,62]. Patients had an average age of 55.8 years and a 1:1 sex distribution. Nearly all patients exhibited primary viral infection symptoms, with the onset of symptoms varying from 24 h to 3 years, possibly because of the latent nature of the virus. Underlying conditions were present in 12 patients, including metabolic disorders such as diabetes [24], impaired glucose tolerance [59], insulin-dependent diabetes [62], and cirrhosis [59]; necessitating immunosuppressive treatment for primary conditions, including kidney transplantation [24], chronic lymphocytic leukemia [62], inflammatory bowel disease [25], autoimmune diabetes [26], and multiple myeloma [61]; ocular disorders, such as herpes zoster ophthalmicus (HZO) with anterior uveitis [58], multifocal choroiditis [27], and viral keratouveitis [27]. Most cases have been treated with antiviral drugs and/or corticosteroids. An unfavorable prognosis includes significant visual acuity decline [62], requiring prolonged medication for secondary choroidal neovascularization and residual scars [26,56].

Zostavax, which contains the Oka strain of VZV, has been linked to some cases, particularly in immunocompromised individuals (Table 1). In three documented cases, patients with different medical histories who received immunosuppressive therapy developed ARN shortly after VZV vaccination [24,25,26]. Two of these patients tested positive for the Oka strain of the VZV vaccine [25,26], suggesting a potential link between vaccine strain infection and ARN. However, VZV DNA tests conducted in seven cases revealed that five patients had wild-type VZV, indicating that most ARN cases may have resulted from VZV reactivation rather than vaccine reactivation. Moreover, the majority of patients had systemic metabolic disorders, such as diabetes, cirrhosis, and inflammatory gastrointestinal diseases, which may contribute to the occurrence of ARN after post-VZV vaccination.

**Figure 3:**
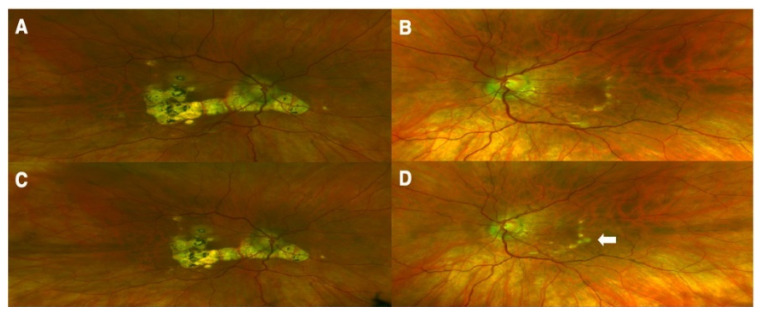
Fundus images of a patient with reactivation of previously controlled uveitis after receiving RZV. Colored fundus photographs of right (**A**) and left (**B**) eyes with chorioretinal scarring due to prior inflammation before RZV vaccination. Photos of stability of the right eye (**C**) and a new active inflammatory lesion in the temporal macula of the left eye after RZV vaccination ((**D**), white arrow). (Adapted with permission from Ref. [27] 2021, Richards et al.).

Recently, a safer alternative to live-strain vaccines has emerged with the approval of the recombinant subunit vaccine RZV (Shingrix, GlaxoSmithKline, Research Triangle Park, NC, USA). This vaccine, which contains the novel adjuvant AS01B, stimulates robust immune responses, potentially inducing persistent cell-mediated immunity [118]. However, adjuvant vaccines such as RZV can increase the likelihood of immune-mediated events, particularly in patients with known inflammatory conditions. To date, only five cases of RZV-associated uveitis have been reported. Heydari-Kamjani et al. reported a case of uveitis sarcoidosis in a 53-year-old female following RZV injection, with initial ocular symptoms occurring only four days after Shingrix vaccination [60]. Chen et al. documented a 65-year-old female with multiple myeloma who developed ARN in her left eye six weeks after RZV vaccination but recovered with valacyclovir and intravitreal foscarnet [61]. More recently, Richards et al. documented three cases of reactivation of previously controlled uveitis after receiving RZV, with various degrees of improvement following antiviral or corticosteroid treatment [27] (Figure 3).

In contrast to the uveitis pathogenesis caused by direct infection from attenuated but still active live attenuated vaccines, there may be two potential mechanisms: (1) the adjuvant AS01B, demonstrated to induce uveitis in various in vitro studies, such as monophosphorylate lipid A (MPL) [118]. (2) Shingrix vaccine production, which involves Chinese hamster ovary (CHO) cells, may result in trace amounts of host cell proteins (HCPs) in the final vaccine product, potentially triggering autoimmune responses [118].

Consequently, vaccination suitability should be rigorously evaluated in patients with underlying inflammatory conditions and those receiving immunosuppressive agents. Live attenuated vaccines may not be the optimal choice for patients with compromised immune function or immunosuppressants. Where possible, PCR testing to determine the VZV DNA type could aid in identifying potential causes of infection and guide targeted therapeutic interventions. Antiviral drugs and corticosteroids have demonstrated efficacy, and vitrectomy may be performed when necessary.

#### 3.2.6. Yellow Fever Vaccine/Hepatitis A Virus (HAV) Vaccine/Co-Administration

Although rarely reported as a uveitis trigger, the yellow fever vaccine, a live attenuated vaccine, has been associated with autoimmune disorders such as neuromyelitis optica spectrum disorder [119]. Only four reports (involving five patients) were included [64,65,66,67], with an average age of 34.4 years and a male-to-female ratio of 3:2. All patients developed symptoms within 3 weeks post-vaccination and initially presented with systemic manifestations. Treatment with local and/or systemic corticosteroids led to improvements. In rare severe adverse reactions, such as yellow fever vaccine-associated viscerotropic and neurological diseases, particular attention is needed. Volkov et al. reported a patient with bilateral acute anterior uveitis and intermediate uveitis secondary to viscerotropic disease after post-vaccination [64]. Initially, severe systemic symptoms included fever, cough, dyspnea, malaise, sore throat, non-bloody diarrhea, trunk maculopapular rash, and yellow fever virus RNA (+). After corticosteroid treatment, the patient improved but experienced persistent fatigue for several months. The mechanism of uveitis after yellow fever vaccination is unknown, with some studies suggesting that immune responses disrupt prior tolerance [65], whereas others propose molecular mimicry-triggered immune cross-reactivity and subsequent autoimmune disease [63].

The hepatitis A virus vaccine (HAVV) effectively prevents hepatitis A, a virus typically transmitted through contaminated water or shellfish consumption. Our study included only one patient diagnosed with left eye MEWDS post-vaccination who recovered spontaneously within six weeks without treatment [67].

Yellow fever and hepatitis A vaccines are frequently administered as prophylaxis in travelers. Our study includes two cases of concurrent hepatitis A and yellow fever vaccine administration. A 33-year-old male received both vaccines, and typhoid vaccine before a trip to Panama (having previously received a single dose of hepatitis A vaccine) [71], he experienced intermittent scotomas and flashes in the right eye, along with systemic symptoms such as fever, headache, and rash. Another patient experienced rapid progressive painless vision loss in the left eye 10 days after co-administration [69]. Both patients showed spontaneous improvement without treatment. Neither report distinctly leans towards any specific vaccine as the causative factor. In conclusion, the use of yellow fever and hepatitis A vaccines as travelers’ immunization choices warrants clinicians’ awareness of the potential for co-administration to induce uveitis.

### 3.3. Different Types of Uveitis Induced by Antiviral Vaccines

In our investigation, anterior uveitis was predominant among patients, most of whom responded favorably to corticosteroid therapy, mirroring findings from a previous meta-analysis [87]. However, the possibility of posterior uveitis could not be ruled out. Our data included 31 patients presenting with posterior uveitis, encapsulating specific syndromes such as APMPPE, MEWDS, ARN, and Harada-like diseases.

#### 3.3.1. Acute Posterior Multifocal Placoid Pigment Epitheliopathy (AMPEE) and Multiple Evanescent White Dot Syndrome (MEWDS)

Both APMPPE and MEWDS are categorized as primary inflammatory diseases of the choroid and retinal vasculature diseases (PICCP) [120,121], typically manifesting as unilateral or bilateral posterior uveitis. We documented seven cases of APMPPE [36,41,46,47,57] and eight cases of MEWDS [30,33,50,67,68,69,70] after vaccination. The age at onset varied between 11 and 31 years, with a male-to-female ratio of 4:3. All cases revealed bilateral involvement, consistent with previous multicenter retrospective studies [122]. Notably, five patients presented with flu-like symptoms [36,41,46,47,57] a few days before visual symptoms. Whether these symptoms directly contribute to APMPPE or are associated with an increased susceptibility remains unclear.

Patients with APMPPE frequently report acute or subacute visual blurring, scotoma, and metamorphopsia. Dilated fundus examination and optical coherence tomography (OCT). alterations in RPE and deep yellow–white placoid lesions were discerned. Indocyanine green angiography commonly reveals hypofluorescent spots [123]. Fluorescein angiography (FA) showed multifocal early hypofluorescence, followed by late hyperfluorescence. After the resolution of active lesions, permanent changes in the RPE pigmentation dispersion led to projection defects; however, late staining or leakage disappeared with time. Among the cases we included, 71.4% of patients exhibited residual RPE changes during the follow-up period [29,36,41,47]. In some cases, choroidal vessels can be observed at the center of the dark areas [120,124].

MEWDS is an idiopathic, self-limiting retinitis that predominantly affects young women and often presents unilaterally. Among the eight patients, ages ranged from 16 to 53 years, with a male-to-female ratio of 2:6, and the majority occurred in females. Common features include multiple small white-yellow lesions in the outer retina and RPE. Fluorescein angiography indicates early multifocal high-fluorescence spots, followed by late staining of the fovea, usually presenting with orange granularity [122]. Subjective symptoms include decreased visual acuity, photosensitivity, scotomas, and visual field defects. Both APMPPE and MEWDS often show widespread white lesions on fundus examination and persistent RPE changes on OCT.

#### 3.3.2. Vogt–Koyanagi–Harada (VKH)

Harada disease is characterized by bilateral posterior uveitis with optic disc swelling, macular edema, and retinal exudates. When combined with Vogt–Koyanagi disease, which primarily involves anterior segment inflammation, it is classified as a VKH disease [125]. VKH is a rare granulomatous inflammatory condition that targets melanocyte-rich tissues such as the choroid, meninges, skin, and hair follicles. Ocular manifestations include bilateral pan-uveitis, serous retinal detachment, and optic disc hyperemia. The disease comprises four consecutive stages: prodromal, acute uveitis, convalescent, and chronic recurrence [126].

Our analysis covered eight relevant studies on Harada-like diseases with a median gap of 11 days between vaccination and symptom emergence, strongly suggesting a correlation between post-influenza vaccination and VKH. All the patients demonstrated bilateral involvement. The pathogenesis of VKH is theorized to involve a T cell-driven autoimmune response against melanocyte antigens, which is closely associated with the HLA-DR4/HLA-DRB104 alleles [78]. We identified three patients exhibiting allele positivity: HLA-DRB10405 positive [23], HLA-DR04 and 07 positive [22], and HLA-DR4 positive [51]. A significant majority of the patients reported relief after corticosteroid therapy.

#### 3.3.3. Acute Retinal Necrosis (ARN)

ARN is rare. According to the American Uveitis Society, it manifests as over one zone of retinal necrosis, progresses gradually if left untreated with antivirals, encompasses occlusive arterial vascular involvement, and induces a pronounced inflammatory response in the anterior chamber and vitreous cavity [127]. VZV is the main cause of ARN, followed by HSV [128].

This review discusses eight cases of ARN, all post-VZV vaccination. The median age was 65 years (range 20–80 years), highlighting ARN’s potential of ARN to affect all age groups, with a predilection for the elderly population. The male-to-female ratio was 5:3. Unlike earlier reports [129,130], bilateral disease was not as common; 75% (six of eight) of patients had unilateral involvement, possibly because of prompt detection and efficient antiviral intervention.

## 4. Discussion

Although uncommon and cause being difficult to prove, there are several reports of adverse ocular reactions to various vaccines [131,132,133]. A recent review of vaccine-induced ocular adverse events for 2010–2020 highlighted ocular adverse effects associated with multiple vaccines, including optic neuritis, uveitis, and retinal inflammations [131]. Additionally, several reports in recent years have explored the link between vaccines and uveitis [87,134,135,136,137,138,139]. A review published by Benage et al. in 2016 identified hepatitis B vaccine (administered alone or with other vaccines) as the leading cause of vaccine-induced uveitis, followed by human papillomavirus virus (HPV), and influenza vaccine [87]. In this systematic review, we summarize the various types of uveitis triggered by different vaccines and provide detailed descriptions of specific uveitis entities. Our findings suggest that post-vaccination uveitis can affect individuals of all age groups, with a potentially higher susceptibility among women. However, due to the varying indications and circumstances of immunization, it is challenging to draw specific conclusions regarding patient sex and age. It is important to note that the vaccines used in these cases included a variety of formulations, reflecting considerations that may differ depending on the year and underlying conditions of the patient.

In contrast to the study by Benage et al. [87], 18 patients with post-vaccination uveitis were recruited from the influenza vaccine group, which appeared to be the most likely cause of uveitis, followed by vaccination with varicella zoster virus and human papillomavirus (HPV). Patients with influenza vaccine-associated uveitis often present with systemic flu-like symptoms. Factors contributing to these adverse effects may include residual activity from attenuated vaccines and molecular mimicry of immune responses. Therefore, when patients resist antiviral therapy and the test results are negative, clinicians should consider the possibility of vaccine-induced immune-related uveitis. Patients with uveitis following VZV vaccination often have severe systemic disease and poor prognosis. Some patients even succumbed to primary diseases during the follow-up period [59], suggesting that individuals with severe underlying or immune system-related diseases may be more susceptible to adverse reactions to VZV vaccination. This may be due to potential susceptibility to attenuated VZV vaccines. Currently, a novel recombinant VZV vaccine (Shingrix) that greatly benefits patients with underlying conditions is available. However, there are still reports of uveitis after vaccination. Future studies should focus on investigating post-Shingrix uveitis cases. In 2014, Holt et al. reported 24 cases of HPV vaccine-related uveitis in females with a median age of 17 years [139]. Similarly, in our study, all patients with HPV-related uveitis were women, suggesting the need to monitor systematic and ocular manifestations following HPV vaccination. Fraunfelder et al. reported 32 cases of HBV-related uveitis in 2010, with 72.7% of the patients experiencing symptoms after the first administration [138]. Unlike our findings, nearly all the patients in their study recovered after treatment without evidence of long-term vaccine-related adverse effects. This discrepancy may be related to variations in follow-up time and vaccine type. Moreover, definitively attributing causality to a specific vaccine in patients receiving combinations or a series of vaccines is challenging. These adverse effects could potentially be related to adjuvant-containing HAV vaccines [71].

A deeper understanding of “rechallenge” can offer compelling clinical evidence. One patient experienced bilateral posterior uveitis three days after the second dose of the HBV vaccine [29]. Improvement occurred following systemic treatment, yet the patient disregarded the medical advice and received a third dose, leading to recurrence of posterior uveitis. This case provides evidence for a causal relationship between uveitis and vaccine administration. Furthermore, the “exacerbation” and “recurrence” of preexisting uveitis following vaccination warrant attention. A patient with anterior uveitis associated with herpes zoster exhibited worsening of the pre-existing condition three weeks after receiving the VZV vaccine [58]. In 2021, Richards et al. [27] reported on three patients with a history of controlled ocular inflammation of varying degrees and locations who experienced disease recurrence after receiving the VZV vaccine. Potential causes include cell-mediated responses to recombinant zoster vaccines (RZV), reactions to residual viral DNA, vaccine failure, and use of immunosuppressants.

Our review also highlights specific uveitis entities, including MEWDS, AMPMEE, VKH, and ARN. MEWDS and APMPPEE are generally associated with favorable prognoses, given their self-limiting course, which often requires no treatment. While there is no evidence suggesting that corticosteroid therapy affects the final visual acuity, it is still recommended in cases of significant macular involvement or systemic complications. Fiorce et al. analyzed the visual prognosis in patients with APMPPEE and found that the probability of achieving a final visual acuity of 20/25 was less than 40% in cases with foveal involvement, whereas the likelihood of visual recovery beyond 20/25 was nearly 90% in patients without foveal damage [140]. The prognosis for most patients with VKH is good, but if left untreated, it can lead to complications such as glaucoma, cataracts, choroidal neovascularization, and retinal atrophy [141]. Patients diagnosed with ARN often have a history of metabolic disease or immunosuppressant use. One study found that nearly 30% of cases exhibited immunodeficiency, correlating with a more severe disease [142]. In addition, all patients with ARN in our review developed this condition after VZV vaccination; however, the mechanism underlying VZV vaccine-related uveitis remains unclear. All patients tested positive for VZV DNA, although only a few studies have investigated genotyping owing to equipment variations. For patients testing positive for wild-type VZV DNA, post-vaccination immunosuppression might reactivate latent VZV. For those testing positive for the VZV Oka strain, this may be related to the attenuated VZV vaccine. However, it is essential to emphasize that this review provides a direction for future research, and that precise conclusions necessitate further validation through fundamental research.

Interestingly, some patients did not fully recover during the posttreatment follow-up period. These cases included those with residual retinal pigment epithelium (RPE) changes [29,36,41,47,50,71], the need for long-term medical treatment due to incomplete recovery from uveitis [31,35], extensive macular scars [32], documented recurrent leakage on fluorescein angiography (FA) [33], no light perception in the left eye [48], irreversible blind spot enlargement [121], severe drops in visual acuity [42,48,62], and the presence of a sunset glow fundus [51,66]. In future studies, we will analyze the long- and short-term side effects of the vaccine in detail.

The mechanisms underlying post-vaccination uveitis remain unclear, but they are believed to share a pathogenesis similar to that of post-infectious uveitis. The four proposed mechanisms for vaccine-induced uveitis include the following. (1) Direct infection: live attenuated vaccines, such as poliovirus, MMR, and yellow fever, may introduce direct infection with attenuated yet active viral strains [134]. (2) Adjuvant-induced syndrome: adjuvant vaccines (especially aluminum-containing vaccines) such as HPV, HBV, and influenza may trigger a syndrome entitled ASIA (autoimmune/inflammatory syndrome induced by adjuvants; Shoenfeld syndrome). Upon injection, these vaccines do not readily dissolve in the extracellular space but instead aggregate at the injection site, forming aluminum conglomerates. This delayed dissolution allows injected aluminum particles to be rapidly captured by immune system cells and transported to various organs, including the brain, where they can induce systemic symptoms [143]. (3) Delayed hypersensitivity reactions: recombinant vaccines such as HBV and HPV may induce delayed hypersensitivity reactions, and the deposition of immune complexes with subsequent complement activation causes uveitis [144]. (4) Cross-reactivity: this occurs when peptide fragments presented to T cells closely resemble conformationally similar peptides of the uvea, triggering an immune response against uveal antigens [145]. Experimental evidence suggests that melanoma-associated molecules present in the choroid can induce ocular inflammation [146].

Despite these findings, our study has certain limitations. While uveitis has been observed after vaccination, it is possible that the international literature predominantly reports only severe cases. This might introduce a reporting bias. Nevertheless, the overall benefits of vaccines remain paramount for global health. Additionally, our study encompasses a significant period, from 1978 to 2023. It is plausible that updates in vaccine products and disparities in vaccine quality among different companies could influence the types of uveitis. However, our study did not delve into these specific details. This limitation presents a pathway for future research, enabling a more in-depth exploration. Further investigation into these specific variables is warranted to gain a nuanced understanding of their impact on uveitis epidemiology.

## 5. Conclusions

This is the first comprehensive study to date that provides a detailed analysis of the various types of uveitis triggered by antiviral vaccines. Through an extensive database search, we identified a strong link with influenza vaccines, followed by VZV and HPV vaccines. Although anterior uveitis is common, conditions such as APMPPE, MEWDS, and VKH are prominent and deserve attention in clinical practice. The efficacy of corticosteroid treatment was evident; however, half of the observed patients did not achieve full recovery, suggesting possible prolonged effects of the vaccine. Future studies should prioritize long-term monitoring and delve more deeply into the vaccine-induced responses.

## Figures and Tables

**Table 1:** Case reports of uveitis following vaccination.

Reference	Diagnosis	Vaccine Type	Age */Gender	Symptoms/Signs/Lab Tests/Medical History	Interval Post-Vaccination ^	Treatment	Outcome/Follow-Up
**Hepatitis B virus (HBV) vaccine**							
**Fried 1987 [28]**	Bilateral posterior uveitis	Recombinant	20/F	Headache	1st: 3 days after the second dose 2nd: 4 days after the third dose	NR	Recurrence due to re-exposure/not reported
**Brézin 1995 [29]**	Bilateral APMPPE	Recombinant	31/M	None	3 days after the fourth dose	NR	A residual paracentral scotoma/9 m
	Bilateral APMPPE	Recombinant	30/M	None	14 days after the third dose	NR	A residual paracentral scotoma OS 4 m
**Baglivo 1996 [30]**	Bilateral MEWDS	Recombinant	23/F	None	24 h after the third dose	NR	None/3 m
**Sood 2019 [31]**	Bilateral VKH (pan-uveitis)	Recombinant	43/M	Hearing loss, tinnitus, integumentary changes.	3 days after the first dose	po/io steroids	VKH with long-term steroids treatment/5 m
**Human papillomavirus (HPV) vaccine**							
**Khalifa 2010 [32]**	Bilateral ampiginous choroiditis	Quadrivalent/recombinant	17/F	None	3 weeks after the first dose	po steroids	Extensive macular scarring remained/3 m
**Ogino 2014 [33]**	MEWDS (left eye)	Bivalent/recombinant	16/F	Throat pain, headache	2 weeks after the second dose	iv steroids and anti-histamine	FA revealed recurrent leakage/2 y
**Chen, Y.-H. 2014 [34]**	Bilateral pan-uveitis	Quadrivalent/recombinant	27/F	Bilateral knee pain, erythematous papules vertigo, and hearing impairment	4 days after the third dose	io/po steroids	None/2 y
**Dansingani 2015 [23]**	Bilateral pan-uveitis and ERD resembling VKH	Quadrivalent/recombinant	20/F	HLA-DRB1*0405(+)	3 weeks after the second dose	po steroids	None/5 m
**Sawai 2016 [35]**	Bilateral anterior uveitis/TINU	Recombinant	14/F	Fever, general malaise, low back pain	4 days after the first dose	io/gtt steroid	long-term steroids treatment for uveitis/3 y
	Bilateral anterior uveitis/TINU	Recombinant	14/F	NR	10 weeks after the third dose	io/gtt steroid	None/NR
**Ye, H. 2020 [22]**	Bilateral posterior uveitis resembling Harada disease	Divalent/recombinant	29/F	HLA-DR04 and 07(+)	7 days after the third dose	io/po steroids	None/4 m
**Kong 2022 [21]**	Bilateral Harada disease-like uveitis(posterior)	Quadrivalent/recombinant	37/F	None	10 days after the third dose	gtt steroids	None/3 m
**Influenza virus vaccine**							
**Hector 1978 [36]**	Bilateral APMPPE	Live attenuated	21/M	Fever, chills, headache	48 h after the first dose	None	Residual RPE changes/6 m
**Blumberg 1980 [37]**	Iritis (OD) and optic neuritis (OS)	Inactivated	27/M	Fever, arthralgias, and myalgias	14 days after vaccination	Systematic/gtt steroids	C3: 91 mg/dL; ESR: 40 mm/h/1 m
**Blanche 1994 [38]**	Bilateral anterior and posterior uveitis	Inactivated	68/F	Fever	48 h after the first dose	gtt steroids	None/3 m
**Gallagher 2009 [39]**	Bilateral VKH (pan-uveitis)	NR	44/F	Tinnitus	1 month after vaccination	iv/po steroids with steroids-sparing and long-term immunomodulation	None/NR
**Wells 2009 [40]**	Bilateral pan-uveitis	NR	70/M	NR	1 days after the first dose	io/gtt steroids	None/3 m
**Mendrinos 2010 [41]**	Bilateral APMPPE	NR	27/M	Flu-like symptoms	14 days after the vaccination	po steroids	Residual RPE changes/3 m
**Tao 2011 [42]**	Posterior uveitis and ERD (right)	Live attenuated (H1N1)	10/M	None	10 days after vaccination	iv/po steroids	None/1 m
	Bilateral posterior uveitis and ERD	Live attenuated (H1N1)	47/F	High fever, bilateral headache	2 days after vaccination	iv/po steroids	Visual acuity: 0.01/NR
**Rothova 2011 [43]**	Bilateral VZV-associated pan-uveitis	Live attenuated (H1N1)	60/M	NR	4 days after vaccination	systematic/io steroids	The intraocular inflammation slowly subsided/NR
**Goyal 2013 [46]**	MEWDS (right eye)	NR	53/M	NR	10 days after vaccination	None	A paracentral scotoma/1 m
**Williams 2015 [45]**	Retinal artery vasculitis (right eye; anterior uveitis)	Live attenuated	78/F	Right-sided headache	8 weeks after vaccination	gtt steroids	None/9 m
**Branisteanu 2015 [47]**	Bilateral APMPEE	NR	18/F	Intermittent headaches	14 days after vaccination	po steroids	Residual RPE changes/5 y
**Manusow 2015 [48]**	Bilateral pan-uveitis with OIS	Live attenuated	49/F	Polyarthritis, fever, tender cervical lymphadenopathy	4 days after vaccination	iv/po steroids	No light perception OS/1 y
	Pan-uveitis with OIS (right)	Live attenuated	57/M	Mild jaundice, confusion and disorientation to place and time/ESR-84(+)	3 days after vaccination	iv/po steroids	LP, 20/50/3 m
**Gonome 2016 [46]**	Bilateral AMPEE and granulomatous uveitis (pan-uveitis)	NR	30/F	Fever, cough, and nausea	17 days after vaccination	Initiate iv/gtt NSAIDs; After the granulomatous uveitis appearance; gtt steroids	None/1 m
**Kim 2016 [49]**	Bilateral VKH (pan-uveitis)	Live attenuated	52/F	Tinnitus	1 month after vaccination	iv/po steroid	None/NR
**Abou-Samra 2019 [50]**	MEWDS (right eye)	NR	27/F	Fever, rash, oral ulcers, arthralgias, headache, or vertigo.	14 days after vaccination	None	Residual RPE changes/8 w
**Murtaza 2022 [51]**	Bilateral VKH (pan-uveitis)	Inactivated	30/M	Headache, tinnitus, and HLA-DR4(+)	2 days after vaccination	io/po steroids	Sunset glow fundus from choroidal depigmentation/6 m
**Measles–Mumps–Rubella (MMR) vaccine**							
**Islam 2000 [52]**	Bilateral anterior uveitis	Live attenuated	12/F	NR	6 weeks after vaccination	gtt steroids	None/1 y
	Bilateral anterior uveitis	Live attenuated	14/M	NR	4 weeks after vaccination	po/gtt steroids	None/NR
**Sedaghat 2007 [53]**	Bilateral pan-uveitis and dermal vasculitis	Live attenuated	17/F	Fever, chills, skin rash and knee arthritis	5 days after vaccination	po/gtt steroids	None/6 m
**Ferrini 2013 [54]**	Anterior uveitis (left eye) with iris heterochromia and cataract	Live attenuated	12 month/F	HLA-B51, rubella IgG (+)	3 months after vaccination	Systematic/gtt/io steroids cataract extraction	None/3 m
**Varicella zoster virus (VZV) vaccine**							
**Esmaeli-Gutstein 1999 [55]**	Anterior and intermediate uveitis (left eye)	Live attenuated	16/F	Generalized vesicular rash	1 week after vaccination	po acyclovir, gtt steroids	None/NR
**Naseri 2003 [56]**	Herpes zoster virus sclerokeratitis and anterior uveitis (left eye)	Live attenuated	9/M	rash in left face, wild-type VZV DNA (+)	3 years after vaccination	po acyclovir/gtt steroids	NR/NR
**Fine 2010 [57]**	Bilateral APMPPE	Live attenuated	11/F	Severe headaches and tinnitus/VZV Ab (+)	10 days after vaccination	po steroids	NR/1 y
**Charkoudian 2011 [24]**	ARN (left eye)	Live attenuated	77/F	VZV DNA (+)/diabetes mellitus	6 days after vaccination	po/iv antiviral drugs, vitrectomy	NR/NR
	Bilateral ARN	Live attenuated	80/M	rash and fever/VZV DNA (+), immunosuppressant use for renal transplantation	2 months after vaccination	po/iv antiviral drugs, io foscarnet, bilateral vitrectomy	NR/NR
**Gonzales 2012 [25]**	Bilateral ARN	Live attenuated	20/M	Oka strain VZV DNA (+)/ immunosuppressant for an inflammatory gastroenteropathy	1 month after vaccination	io foscarnet, antiviral drugs, pars plana vitrectomy	NR/NR
**Sham 2012 [58]**	Exacerbation of anterior uveitis (right eye)	Live attenuated	86/M	Medical history of HZO with anterior uveitis	3 weeks after vaccination	po Valacyclovir, gtt steroids	None/NR
**Heath 2017 [26]**	ARN (left eye)	Live attenuated	78/F	Oka strain VZV DNA (+)/immunosuppressant for autoimmune diabetes	6 weeks after vaccination	po valaciclovir, gtt steroids, pars plana vitrectomy	A pigmented scar/NR
**Weinlander 2019 [59]**	ARN (left eye)	Live attenuated	64/M	Wild-type VZV DNA (+)/metabolic syndrome and impaired glucose tolerance	16 months after vaccination	po Valacyclovir, po/gtt steroids	None/6 m
	ARN (left eye)	Live attenuated	62/M	Wild-type VZV DNA (+)/Cirrhosis and diabetes mellitus type 2	7 months after vaccination	po Valacyclovir, gtt steroids	Died from complications of his cirrhosis/6 m
**Heydari-Kamjani 2019 [60]**	Bilateral uveitis sarcoidosis	Recombinant zoster vaccine	53/F	Headaches	4 days after vaccination	gtt steroids	None/NR
**Chen R.I. 2020 [61]**	ARN (left eye)	Recombinant zoster vaccine	65/F	Immunomodulator for multiple myeloma/wild-type VZV DNA (+)	6 weeks after vaccination	io foscarnet, iv/po antiviral drugs	None/19 w
**Menghini 2021 [62]**	ARN with obliterative angiopathy (left eye)	Live attenuated	76/M	Insulin-dependent diabetes mellitus, chronic lymphocytic leukemia/wild-type VZV DNA (+)	2 days after vaccination	io foscarnet, iv/po/iv antiviral drugs, iv/po steroids	Left eye visual acuity dropped to perception only/NR
**Richards 2021 [27]**	Recurrent bilateral multifocal choroiditis	Recombinant zoster vaccine	57/F	arm swelling at the injection site, chills, malaise, subjective fever, and tinnitus/immunosuppressant for multifocal choroiditis	24 h after the first dose	po steroids and continued methotrexate	Intravitreal bevacizumab for a secondary choroidal neovascular membrane/2 m
	Recurrent bilateral anterior and mild intermediate uveitis	Recombinant zoster vaccine	69/M	Headache/gtt steroids for uveitis	1 month after the second dose	po valacyclovir/gtt steroids	None/1 m
	Recurrent anterior uveitis (left eye)	Recombinant zoster vaccine	70/F	gtt steroids and po valacyclovir for viral keratouveitis	2 weeks after the first dose	po valacyclovir, po/gtt steroids	None/6 w
**Yellow Fever virus vaccine**							
**Biancardi 2019 [63]**	Anterior uveitis (right eye)	Live attenuated	35/F	None	10 days after vaccination	gtt steroids	None/NR
	Intermediate uveitis (left eye)	Live attenuated	21/F	low fever, body ache, and mild headache	14 days after vaccination	po steroids	None/6 w
**Volkov 2020 [64]**	Viscerotropic disease followed by bilateral acute anterior and intermediate uveitis	Live attenuated	37/M	Fever, cough, dyspnea, malaise, sore throat, non-bloody diarrhea, and morbilliform skin rash of the chest/YFV RNA (+)	2–3 weeks after vaccination	io/gtt steroids	Persistent fatigue for few month/NR
**Campos 2021 [65]**	Bilateral VKH (pan-uveitis)	Live attenuated	34/M	tinnitus, headache	12 days after a booster dose	Iv/po steroids	None/2 y
**Pereima 2022 [66]**	Bilateral Acute VKH (pan-uveitis)	Live attenuated	45/M	tinnitus, headache	2 weeks after vaccination	Iv/po steroids	Sunset glow fundus and dark dots/30 m
**Hepatitis A virus (HAV) vaccine**							
**Fine 2001 [67]**	MEWDS (left eye)	Inactivated	30/M	Not reported	13 days after booster vaccination	None	None/6 w
**Rabies vaccine**							
**Yang 2018 [68]**	MEWDS (left eye)	Inactivated	33/F	None	7 days after the third dose	io steroids	None/3 y
**Co-administration**							
**Stangos 2006 [69]**	MEWDS (left eye)	HAV/yellow fever	50/F	None	1 week after vaccination	None	None/6 w
**Cohen 2010 [70]**	MEWDS (left eye)	HPV/Meningococcus	17/F	HLA-B27(+)	1 months after vaccination	None	None/2 m
**Escott 2013 [71]**	Acute multifocal choroiditis (right eye)	HAV/typhoid/yellow fever	33/M	fever, rash, oral ulcers, arthralgias, headache, vertigo	3 weeks after vaccination	None	RPE atrophy/8 w

* In years unless otherwise listed. ^ Time between last vaccination and initial ocular symptom/sign. HBV = hepatitis B virus; APMPPE = acute posterior multifocal placoid pigment epitheliopathy; MEWDS = multiple evanescent white dot syndrome; VKH = Vogt–Koyanagi–Harada; HPV = human papillomavirus; TINU = tubulointerstitial nephritis and uveitis; MMR = measles–mumps–rubella; VZV = varicella zoster virus; HAV = hepatitis A virus; OIS = orbital inflammatory syndrome; OS = left eye; OD = right eye; ERD = exudative retinal detachment; ESR = erythrocyte sedimentation rate; ARN: acute retinal necrosis; NSAIDs = non-steroid anti-inflammatory drugs; RPE = retinal pigment epithelium; HLA = human leukocyte antigen; io = intra-orbital/retrobulbar; iv = intravenous; gtt = eyedrops; po = orally; NR = not reported; Ab = antibody; HZO = herpes zoster ophthalmic; FA = fluorescein angiography.

## Data Availability

All data related to this study are presented and published here.
